# Gene augmentation of *LCA5*-associated Leber congenital amaurosis ameliorates bulge region defects of the photoreceptor ciliary axoneme

**DOI:** 10.1172/jci.insight.169162

**Published:** 2023-05-22

**Authors:** Siebren Faber, Olivier Mercey, Katrin Junger, Alejandro Garanto, Helen May-Simera, Marius Ueffing, Rob W.J. Collin, Karsten Boldt, Paul Guichard, Virginie Hamel, Ronald Roepman

**Affiliations:** 1 Department of Human Genetics, Research Institute for Medical Innovation, Radboud University Medical Center, Nijmegen, Netherlands.; 2 Department of Molecular and Cellular Biology, University of Geneva, Geneva, Switzerland.; 3 Division of Experimental Ophthalmology and Medical Proteome Center, Institute for Ophthalmic Research, Eberhard Karls University Tübingen, Tübingen, Germany.; 4 Department of Pediatrics, Research Institute for Medical Innovation, Radboud University Medical Center, Nijmegen, Netherlands.; 5 Cilia Cell Biology, Institute of Molecular Physiology, Johannes-Gutenberg University, Mainz, Germany.; 6 Department of Human Genetics, Donders Institute for Brain, Cognition and Behaviour, Radboud University Medical Center, Nijmegen, Netherlands.

**Keywords:** Cell Biology, Genetics, Gene therapy, Molecular genetics, Retinopathy

## Abstract

Leber congenital amaurosis (LCA) is a group of inherited retinal diseases characterized by early-onset, rapid loss of photoreceptor cells. Despite the discovery of a growing number of genes associated with this disease, the molecular mechanisms of photoreceptor cell degeneration of most LCA subtypes remain poorly understood. Here, using retina-specific affinity proteomics combined with ultrastructure expansion microscopy, we reveal the structural and molecular defects underlying LCA type 5 (LCA5) with nanoscale resolution. We show that *LCA5*-encoded lebercilin, together with retinitis pigmentosa 1 protein (RP1) and the intraflagellar transport (IFT) proteins IFT81 and IFT88, localized at the bulge region of the photoreceptor outer segment (OS), a region crucial for OS membrane disc formation. Next, we demonstrate that mutant mice deficient in lebercilin exhibited early axonemal defects at the bulge region and the distal OS, accompanied by reduced levels of RP1 and IFT proteins, affecting membrane disc formation and presumably leading to photoreceptor death. Finally, adeno-associated virus–based *LCA5* gene augmentation partially restored the bulge region, preserved OS axoneme structure and membrane disc formation, and resulted in photoreceptor cell survival. Our approach thus provides a next level of assessment of retinal (gene) therapy efficacy at the molecular level.

## Graphical abstract

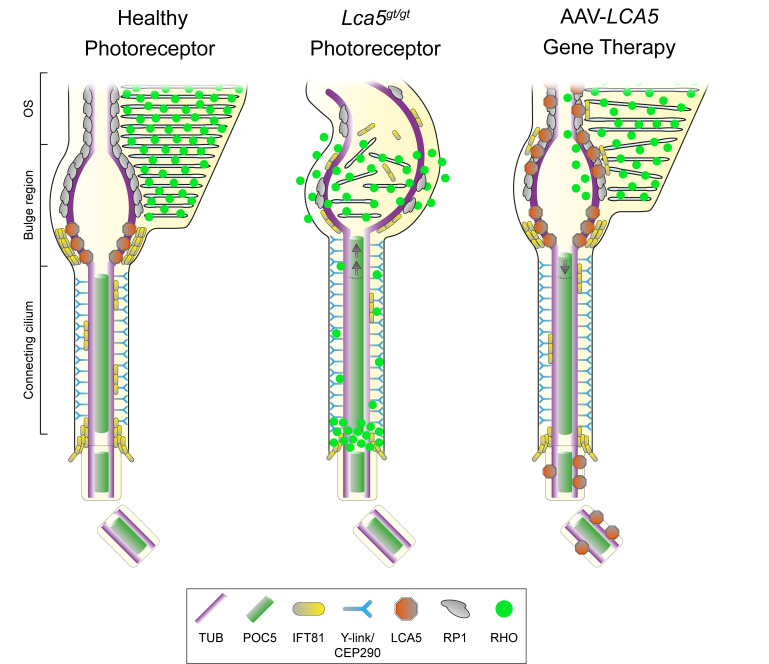

## Introduction

Mutations in the *LCA5* gene disrupt its cognate ciliary protein lebercilin, causing Leber congenital amaurosis (LCA), one of the most severe forms of inherited blindness (1). Phenotypically, LCA manifests as nystagmus, delayed pupillary responses, photophobia, hyperopia, and severely decreased visual acuity, in the first year of life (2). The cells that are primarily affected in patients with LCA5 are the light-sensing photoreceptors, the most abundant cell type in the human retina (1, 3). Photoreceptors are highly specialized neuroepithelial cells, consisting of structurally and physiologically distinct cellular compartments, including a synaptic terminal, an inner segment (IS), an outer segment (OS), and a connecting cilium (CC) that bridges the IS and OS (4).

Lebercilin has been shown to be widely expressed in human and mouse tissues, notably at the level of ciliated epithelia (1, 5, 6). However, despite this ubiquitous pattern of expression, *LCA5* mutations lead to a retina-restricted phenotype in humans, mice, and zebrafish (1, 7, 8), suggesting a crucial function of this protein in the retina. In the mouse eye, *Lca5* mRNA is increasingly expressed in photoreceptor cells during development (1), arguing for a specific role of lebercilin in these cells. At the subcellular level, lebercilin has been described as a microtubule-binding protein, which localizes at the base of the primary cilium in epithelial cells (1, 7). In line with this, lebercilin interacts with several centriole/basal body and ciliary components such as oral-facial-digital syndrome 1 protein (9), ninein-like protein (10), or intraflagellar transport (IFT) core proteins (7). Based on regular immunohistochemistry, lebercilin has been proposed to localize to the photoreceptor CC, a highly developed equivalent of the ciliary transition zone involved in selective transport of OS proteins, lipids, and membrane vesicles (1, 7, 11, 12). Recently, we optimized super-resolution ultrastructure expansion microscopy (U-ExM) for retinal imaging to get further insights into the molecular architecture of photoreceptor OS with nanoscale resolution. This technique, based on the isotropic physical expansion of a biological sample embedded in a swellable gel, revealed that lebercilin localizes to a specific photoreceptor region, which we called “bulge region,” apical to the CC (13), in accordance with our previous electron microscopy analysis (1). This region exhibits a stereotypical enlargement of the axonemal microtubules compared with the CC, correlating with the absence of the inner scaffold, a recently described molecular zipper maintaining cohesion between microtubule doublets (MTDs) at the level of the CC and the centrioles (13, 14). Currently, there is not much known about the exact function of the bulge region. It is suggested that it is important for the initiation of OS membrane disc formation in an actin-dependent manner, mediated by photoreceptor cilium actin regulator (PCARE) and Wiskott-Aldrich syndrome protein family member 3 (WASF3) (15). Given the retina-specific phenotype observed with mutations of *LCA5*, unraveling the function of the bulge protein lebercilin could bring insights into the role of the bulge region in context of specific forms of inherited retinal diseases (IRDs).

In addition to deciphering the molecular mechanisms associated with IRDs, one of the most exciting challenges in recent years in the ophthalmologic field is the development of therapeutic strategies to restore vision in the context of these diseases, which are to date mostly incurable. Gene augmentation therapy has emerged as an attractive tool for monogenic IRDs, because the eye is easily accessible for delivery injection techniques, immune privileged, and highly suitable for assessing functional outcomes (16–20). Commonly, delivery of a gene therapy is performed by intravitreal or subretinal injection of a functional copy of the affected gene, encapsulated by a viral vector (21). Now, Luxturna is the only FDA-approved gene therapy amenable to LCA with biallelic *RPE65* mutations, though several other gene therapies are currently in clinical trials (21, 22). Before getting to the clinical phase, preclinical animal studies are essential for the development of safe and effective gene therapies (21). The quality of the outcome measures of these animal studies are therefore of utmost importance to enhance the success rate in later stages. The methods that are used to assess the therapeutic efficacy of the gene therapy in most preclinical gene therapy studies are mainly focused on the assessment on tissue level and visual acuity, including electroretinography, optical coherence tomography, and retinal immunostainings (23–26). Although these methods are valuable tools to assess gene therapy efficacy in mice, we are currently lacking assays to evaluate the efficacy of gene augmentation therapy at the photoreceptor cellular and ultrastructural level. Here, we propose a potentially novel and easily accessible approach based on U-ExM to tackle this question at a nanoscale resolution, using a previously described gene therapy for LCA5 (23, 24) as proof of concept.

## Results

### Affinity proteomics of lebercilin in mouse retina reveals photoreceptor-associated modules.

To better understand the molecular mechanisms leading to LCA, we first investigated the retina-specific interactome of lebercilin in both homozygous *Lca5* gene trap (*Lca5^gt/gt^*; HOM) and heterozygous *Lca5^+/gt^* (HET) mice. Hence, we used an adeno-associated virus 7m8 (AAV7m8) vector to deliver a human *LCA5* cDNA construct (23, 24), encoding lebercilin fused at the C-terminus with a 3xFLAG tag, into the eyes of the mice at P2 by intravitreal injection. Four weeks postinjection, anti-Flag affinity purification was performed followed by mass spectrometry analysis.

**Figure 1:**
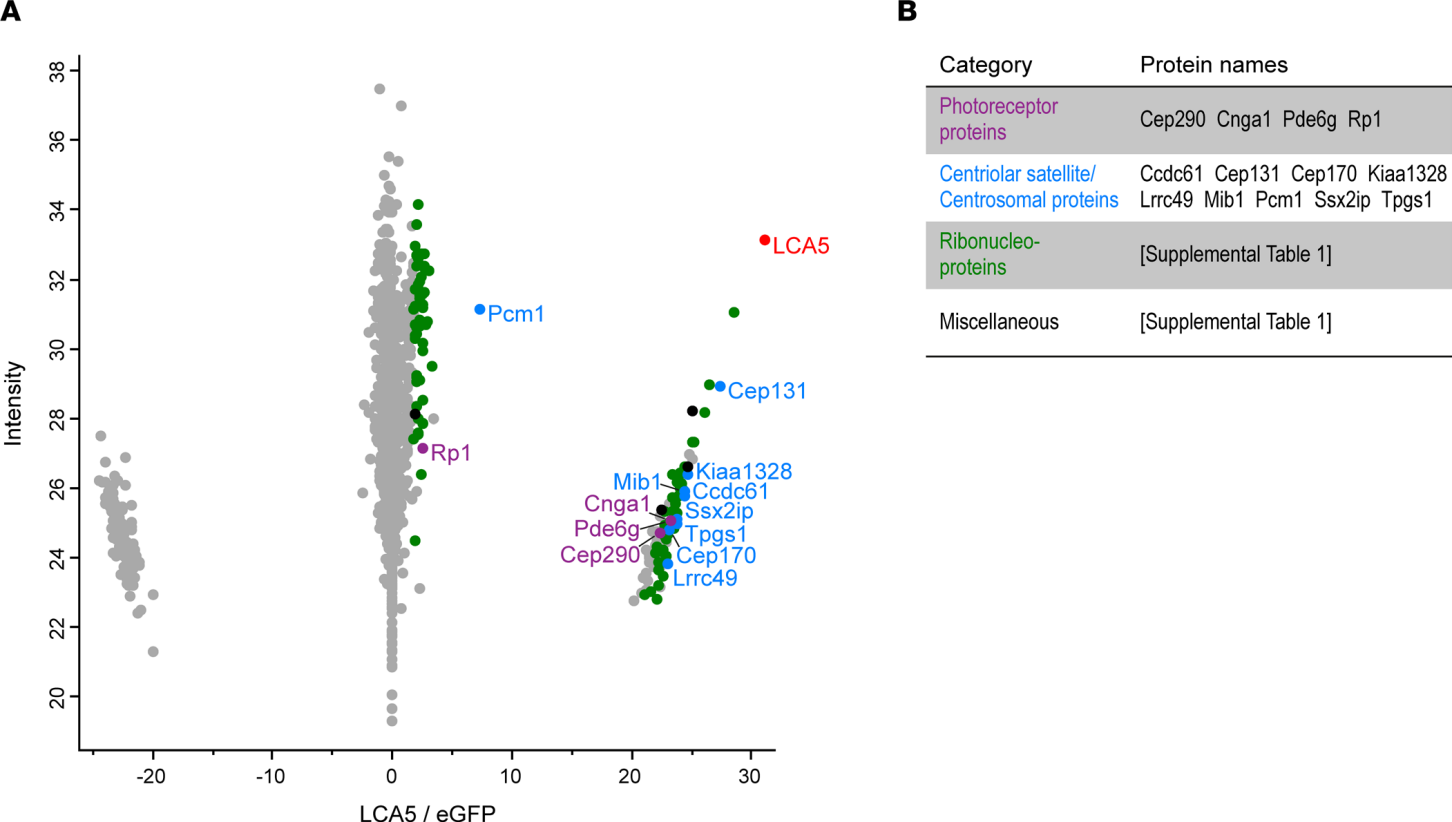
Identification and clustering of potential lebercilin interactors. (**A**) Scatterplot showing enriched proteins, comparing AAV-*LCA5*–injected retinas to AAV-*eGFP*–injected (control) retinas in *Lca5^gt/gt^* (HOM) mice. The bait protein lebercilin (LCA5) is shown in red. Significantly enriched proteins (*P* < 0.05 by Student’s *t* test and FDR < 0.05 by significance A test) are categorized into different groups based on their function, including photoreceptor-associated proteins (purple), centriolar satellite/centrosomal proteins (blue), ribonucleoproteins (green), and miscellaneous (black). *X* axis represents log_2_ ratio between AAV-*LCA5*–injected and AAV-*eGFP*–injected (control) retinas. *Y* axis represents the intensity score, indicating the relative amounts of proteins in the data set. There were 15 mice per biological replicate (*n* = 5). (**B**) Table showing the significant proteins categorized in different groups, based on their function. Original data are listed in Supplemental Table 1.

Analysis of the lebercilin-associated proteins in injected HOM mice revealed a total of 108 significantly enriched proteins (Figure 1A and Supplemental Table 1; supplemental material available online with this article; https://doi.org/10.1172/jci.insight.169162DS1). Analysis of the lebercilin-associated proteins in injected HET mice showed a highly similar set of significant proteins (Supplemental Figure 1 and Supplemental Table 1), validating the robustness of our approach.

The identified significant proteins can be categorized into different groups based on their functions. These groups include photoreceptor-associated proteins, centriolar satellite/centrosomal proteins, ribonucleoproteins, and miscellaneous (Figure 1B, Supplemental Figure 1B, and Supplemental Table 1).

Since lebercilin is a ciliary protein with a prominent role in photoreceptors (1, 7), we focused on the photoreceptor-associated proteins and the centriolar satellite/centrosomal proteins identified in our data sets (Figure 1, A and B). More specifically, we further investigated the central player in the centriolar satellite/centrosomal proteins group, pericentriolar material 1 protein (PCM1) (27, 28), and 2 photoreceptor-associated proteins, centrosomal protein of 290 kDa (CEP290) and retinitis pigmentosa 1 protein (RP1). Interestingly, we recently found that lebercilin localizes in the extension of CEP290 (13), suggesting an interdependence between these proteins, and mutations in *CEP290* also lead to LCA (29). Similarly, RP1 has been shown to localize at the distal part of the CC (30, 31), similar to lebercilin, and mutations in *RP1* lead to retinitis pigmentosa (32, 33).

### Lebercilin is a bulge protein localizing in between CEP290 and RP1.

**Figure 2:**
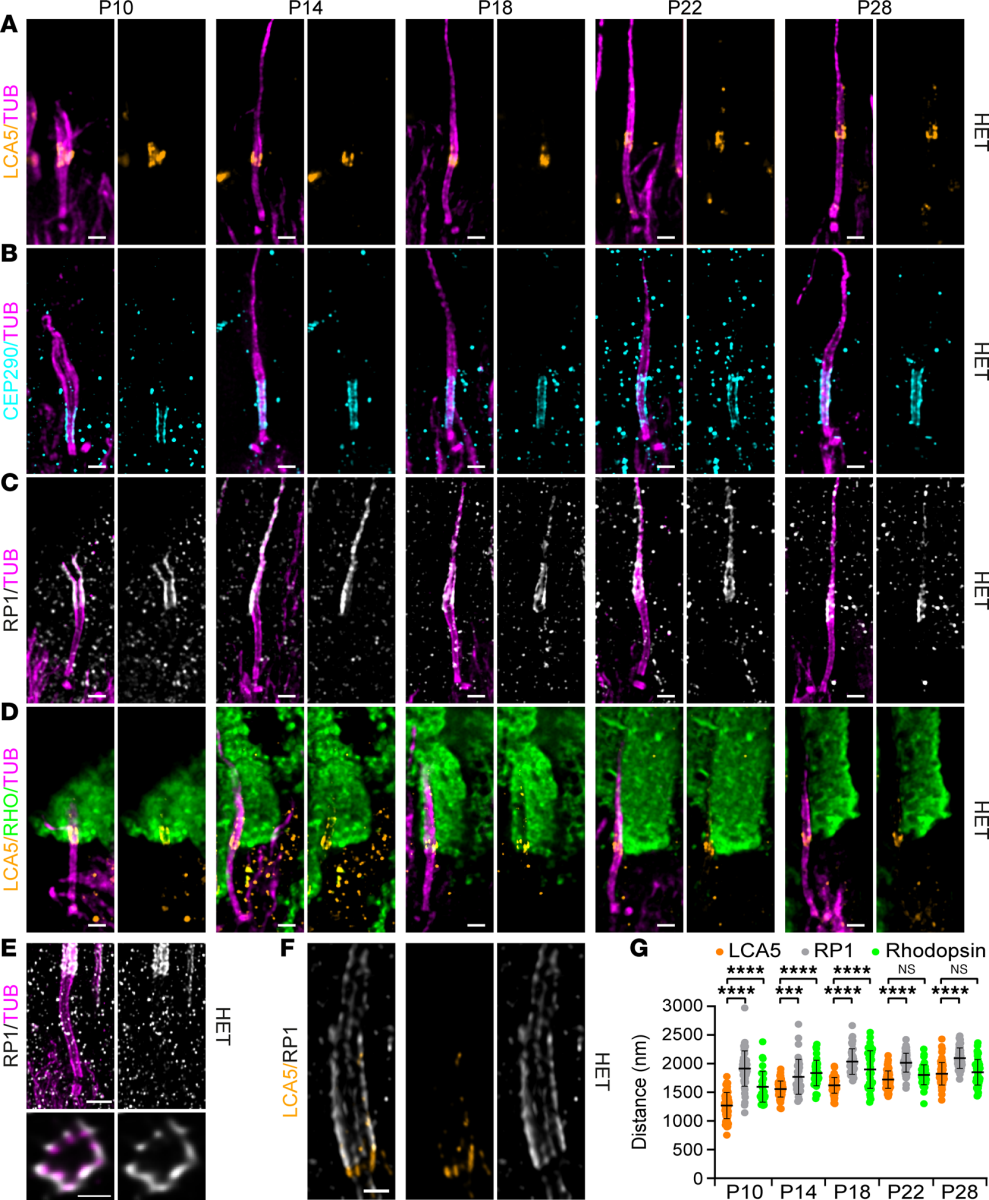
Nanoscale mapping of lebercilin. (**A**–**D**) Widefield (original magnification, 63×) images of expanded photoreceptors stained for tubulin (magenta) and lebercilin (LCA5; orange, **A**), CEP290 (cyan, **B**), RP1 (white/gray, **C**), or LCA5/rhodopsin (orange/green, **D**) from P10 to P28 in *Lca5^+/gt^* (HET) mice. Scale bars: 500 nm. (**E**) Confocal U-ExM images of adult photoreceptor stained for tubulin (magenta) and RP1 (white/gray). Lower panels show transversal view of the bulge region. Scale bars: 500 nm (side view), 200 nm (transversal view). (**F**) Confocal U-ExM image of adult photoreceptor stained for LCA5 (orange) and RP1 (white/gray). Scale bar: 200 nm. (**G**) Quantification of the distance of LCA5 (orange), RP1 (white/gray), and rhodopsin (green) signal proximal ends to the mother centriole proximal end from P10 to P28. Three animals per time point. Data presented as mean ± SD; *n* = 35–80. ***P < 0.001, ****P < 0.0001 by Kruskal-Wallis test with Dunn’s multiple-comparison test.

To precisely map these potential lebercilin interactors in photoreceptor cells at high resolution, we exploited super-resolution U-ExM, which we recently optimized for expanding mouse retinal tissue (13). Consistent with our previous work (13), we found lebercilin predominantly at the proximal part of the bulge region, with an additional weaker signal detected at the distal axoneme and the centrioles (Figure 2A). Since lebercilin shows faint localization at the centrioles, we first investigated the photoreceptor-specific localization of the centriolar satellite protein PCM1, which is also involved in the correct localization of several proteins to the centrioles (34). As expected, we found that PCM1 localizes to the centriolar satellites, but we could not detect it at the bulge region (Supplemental Figure 2A), suggesting that lebercilin and PCM1 might interact at the level of satellites rather than along the photoreceptor axoneme. To address this point, we costained PCM1 and lebercilin in human U2OS cultured cells (Supplemental Figure 2B) and found that both colocalized at the level of centriolar satellites.

Next, we set out to investigate the specific location of CEP290 and RP1 in the developing photoreceptors from P10 until P28 in HET mice. CEP290 was consistently detected at the distal end of the daughter centriole and the external part of the microtubules along the CC (Figure 2B), verifying that lebercilin localizes in the extension of CEP290 (13). Measuring the lengths of CEP290 and centrosomal protein POC5 along the CC showed that their signal gradually increased during photoreceptor development, reaching a plateau phase from P18 until P28, with an average final length of 1,420 nm (CEP290) and 1,552 nm (POC5), similar to what we previously reported (13) (Figure 2B and Supplemental Figure 3B). Interestingly, we found that RP1 localized to the distal part of the bulge region, where it displays a stereotypical 9-fold symmetry on the external part of the MTDs similarly to lebercilin (13) and also extends toward the distal axoneme (Figure 2, C and E). Moreover, costainings of lebercilin and RP1 revealed that RP1 was continuous to the lebercilin signal (Figure 2F). We next mapped its position relative to lebercilin by measuring the distance from the centriolar distal end to the proximal end of the RP1 and lebercilin signals. We demonstrated that the RP1 signal was significantly higher compared with lebercilin at all time points (Figure 2G). Remarkably, the RP1 signal distance was significantly longer at P10 compared with P14 (Figure 2G; *P* = 0.0005), possibly explained by the immaturity of the axoneme at P10, including the bulge region. Previously, it was shown that RP1 regulates distal axoneme formation and is required for proper OS formation (30, 35). Therefore, we investigated the localization of the initiation site of the OS by staining the OS marker rhodopsin relative to lebercilin. We found that lebercilin localizes next to the proximal extremity of the rhodopsin staining (Figure 2D). By measuring the proximal end distance of the rhodopsin signal, we found that it was above the proximal signal of lebercilin at P10, P14, and P18, while at P22 and P28 it coincided with the proximal signal of lebercilin (Figure 2G). This suggests that the bulge region might indeed be crucial for OS membrane disc formation.

### Lebercilin loss affects CC length, bulge formation, and distal axoneme organization.

To better understand the role of the bulge region in photoreceptor development, and more specifically the function of lebercilin at this location, we made use of an *Lca5^gt/gt^* (HOM) mouse model (7). Phenotypically, this mouse model fully recapitulates LCA in humans, showing an early-onset, progressive retinal degeneration, with only a few layers of photoreceptor nuclei left in the outer nuclear layer (ONL) at P28 (7). It is noteworthy that assessment of the P28 time point in HOM retinas is rather difficult, since most photoreceptors, and especially their distal axonemes, are already lost at this time point.

**Figure 3:**
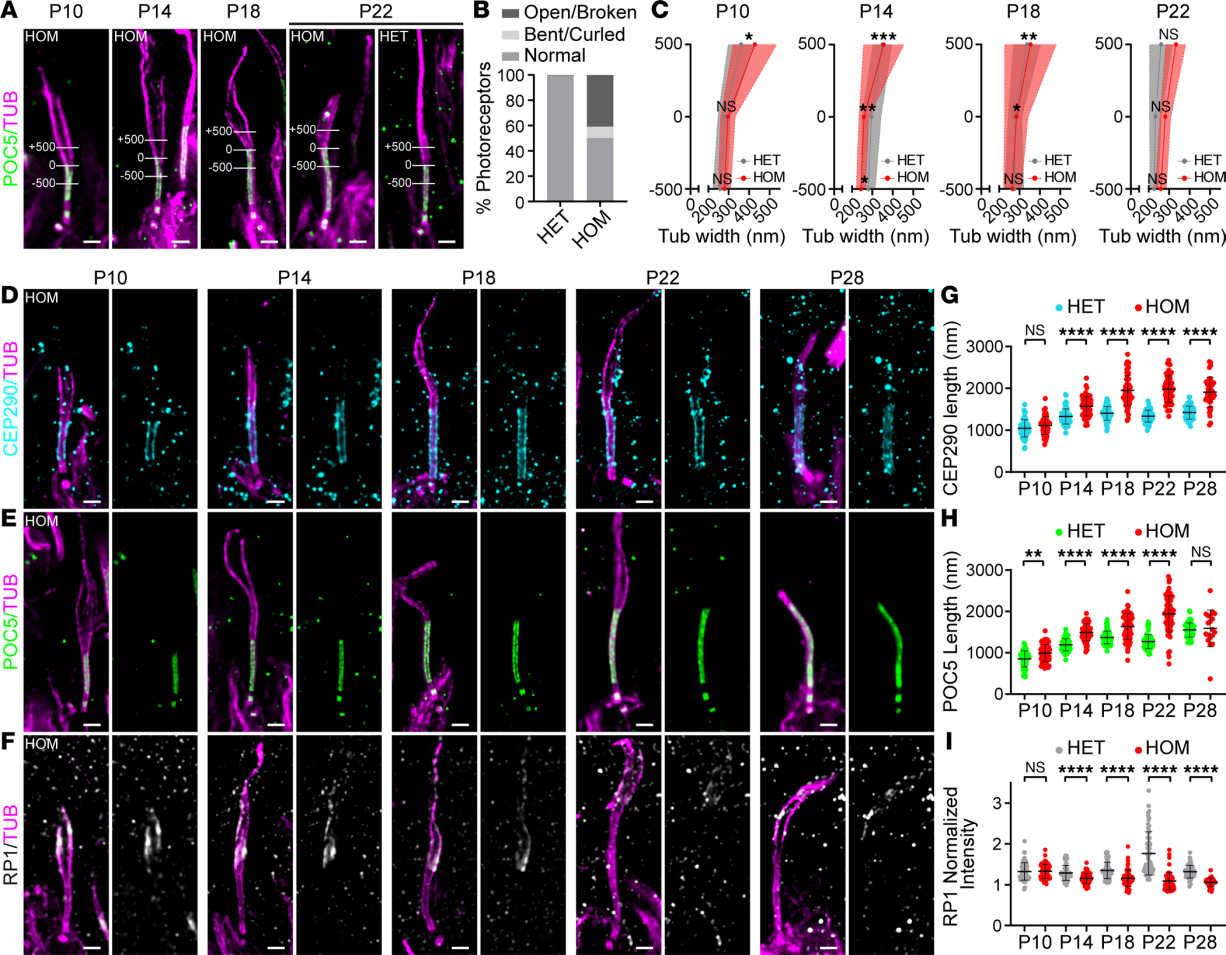
Effect of lebercilin loss on bulge formation, distal axoneme organization, and CC length. (**A**) Widefield (original magnification, 63×) images of expanded photoreceptors stained for POC5 (green) and tubulin (magenta) from P10 to P22 in *Lca5^gt/gt^* (HOM) mice and in P22 *Lca5^+/gt^* (HET) mice. Lines in images illustrate the measurements used in **C** of tubulin width at 3 locations: +500 nm, 0 nm, −500 nm. The distal end of CC inner scaffold marker POC5 was used to set the 0 location. Scale bars: 500 nm. (**B**) Distal axoneme (above CC) conformations of HET versus HOM photoreceptors at P18 and P22 indicated in percentages. Photoreceptor distal axoneme conformations: normal (HET: 99.6%; HOM: 50.2%), open/broken (HET: 0.4%; HOM: 40.8%), and bent/curled (HET: 0%; HOM: 9%). *n* = 247 (HET), *n* = 217 (HOM). (**C**) Tubulin width measurements of the photoreceptor at the 3 locations depicted in **A** from P10 to P22. Average tubulin width at each location is indicated by a gray or red dot for HET and HOM, respectively. P28 not included, since most of the distal axonemes are lost at this time point. Only photoreceptors that were stained for tubulin and POC5 were used for the measurements. Three animals per time point. Data presented as mean ± SD; *n* = 16–29. *P < 0.05, **P < 0.01, ***P < 0.001 by *F* test. Significance represents the tubulin width dispersion between HET and HOM. (**D**–**F**) Widefield (original magnification, 63×) images of expanded photoreceptors stained for tubulin (magenta) and CEP290 (cyan, **D**), POC5 (green, **E**), or RP1 (white/gray, **F**) from P10 to P28 in HOM mice. Scale bars: 500 nm. (**G**–**I**) Impact of lebercilin loss on CEP290 length (**G**), CC inner scaffold length (POC5, **H**), or RP1-normalized intensity at the bulge region (**I**) from P10 to P28. Three animals per time point. Data presented as mean ± SD; *n* = 39–65 (**G**), *n* = 19–60 (**H**), *n* = 39–72 (**I**). **P < 0.01, ****P < 0.0001 by Mann-Whitney test.

First, we looked at the impact on photoreceptor integrity by staining the axonemal microtubules using U-ExM in HET versus HOM mice. We found that approximately 50% of photoreceptors of HOM mice show distal axoneme abnormalities with a loss of cohesion of the microtubules above the CC, including open/broken and bent/curled conformations (Figure 3, A and B). To validate the observed loss of cohesion in the distal axoneme, we measured the axoneme diameter at different positions: at the distal end of the CC (0 nm), 500 nm below the CC (−500), and 500 nm above the CC (+500) in both HET and HOM mice (Figure 3, A and C). We found a significant increase in axoneme diameter above the CC (+500 nm) in HOM mice at P10 (*P* = 0.0468) and P22 (*P* < 0.0001), but not at P14 (*P* = 0.6842) and P18 (*P* = 0.7434). However, we did find a significantly increased dispersion of tubulin width in the HOM photoreceptors at P10, P14, and P18 compared with HET mice (Figure 3C), suggesting that loss of lebercilin leads to the collapse or the spreading of microtubules above the CC.

Based on the tubulin staining, the CC seemed unaffected by the loss of lebercilin. To verify this, we stained for the CC proteins CEP290 and POC5 and found that they were properly localized in the absence of lebercilin (Figure 3, D and E). However, when measuring the lengths of the CEP290 or POC5 signal along the CC, we found them to be significantly increased from P14 to P28 (Figure 3, G and H), indicating that the overall CC length increases in the absence of lebercilin and of the bulge region.

Since the distal axoneme is clearly disrupted by the loss of lebercilin, we hypothesized that RP1 will also be affected. Indeed, we could show that in the absence of lebercilin, RP1 levels at the bulge region, while not yet affected at P10, were significantly decreased at almost every time point, with almost no signal left at P28 (Figure 3, F and I).

### Lebercilin loss affects rhodopsin localization.

**Figure 4:**
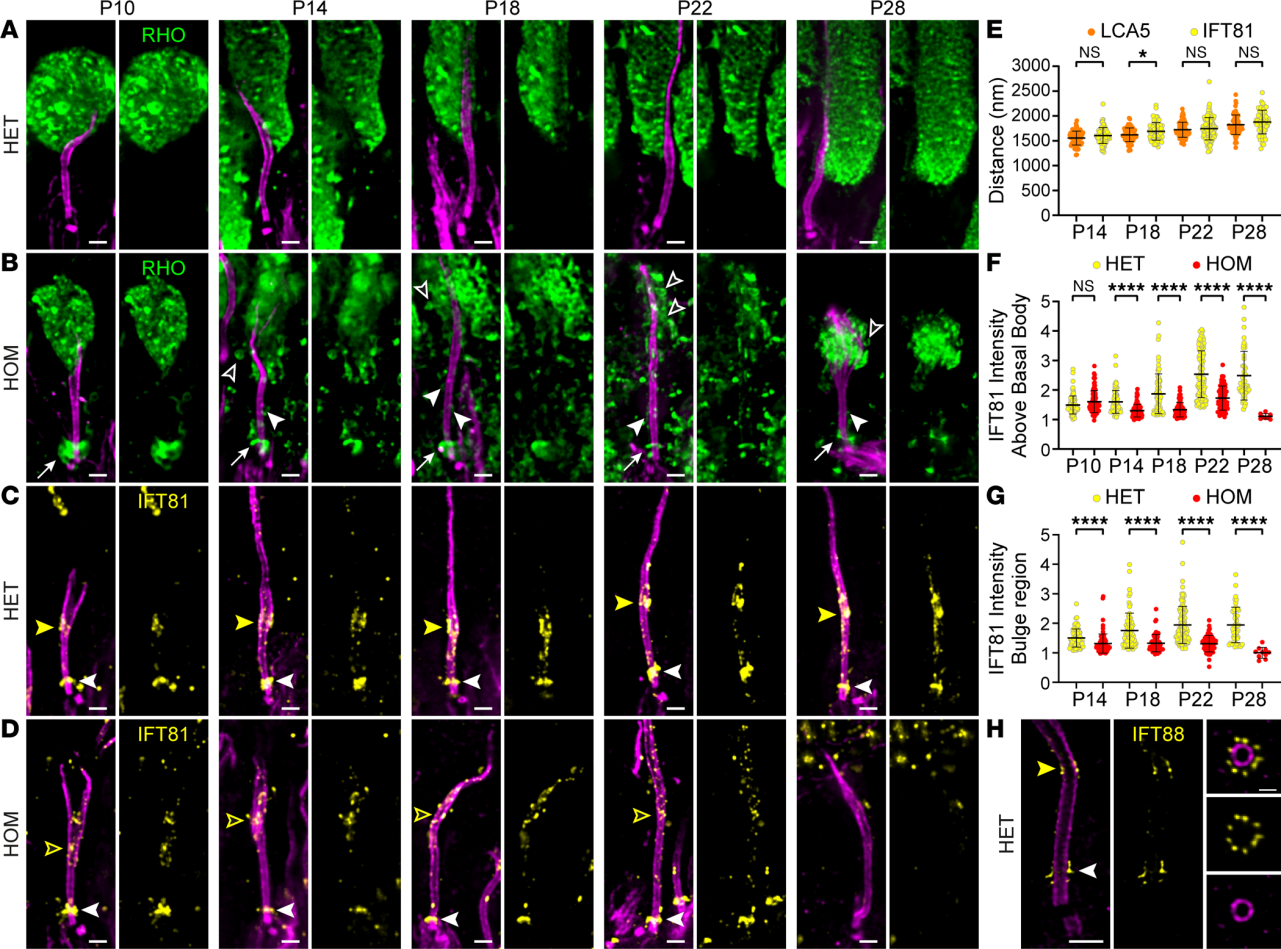
Effect of lebercilin loss on OS formation and intraflagellar transport. (**A** and **B**) Widefield (original magnification, 63×) images of expanded photoreceptors stained for tubulin (magenta) and rhodopsin (green) from P10 to P28 in *Lca5^+/gt^* (HET) (**A**) and *Lca5^gt/gt^* (HOM) (**B**) mice. White arrows indicate accumulation of rhodopsin above the basal body. White arrowheads indicate rhodopsin along the CC. Open white arrowheads indicate rhodopsin in vesicle-like structures. Scale bars: 500 nm. (**C** and **D**) Widefield (original magnification, 63×) images of expanded photoreceptors stained for tubulin (magenta) and IFT81 (yellow) from P10 to P28 in HET (**C**) and HOM (**D**) mice. White arrowheads indicate IFT81 localization above the basal body. Closed and open yellow arrowheads indicate IFT81 localization at the bulge region in HET and HOM, respectively. Scale bars: 500 nm. (**E**) Quantification of the distance of lebercilin (LCA5; orange; same values as in Figure 2G) and IFT81 bulge region (yellow) signal proximal ends to the mother centriole proximal end from P14 to P28. P10 not included, since the bulge region is not properly formed yet at this time point. Three animals per time point. Data presented as mean ± SD; *n* = 62–113. *P < 0.05 by Mann-Whitney test. (**F** and **G**) Impact of LCA5 loss on IFT81-normalized intensity above the basal body (**F**) or at the bulge region (**G**) from P10 to P28. P10 not included for IFT81 intensity at the bulge region, since it is not properly formed yet at this time point. Three animals per time point. Data presented as mean ± SD; *n* = 8–113 (**F**), *n* = 10–113 (**G**). ****P < 0.0001 by Mann-Whitney test. (**H**) Confocal U-ExM images of adult photoreceptor stained for tubulin (magenta) and IFT88 (yellow). Right panels show transversal view of the bulge region. White arrowhead indicates IFT88 localization above the basal body. Yellow arrowhead indicates IFT88 localization at the bulge region. Scale bars: 500 nm (side view), 200 nm (transversal view).

We showed that lebercilin localized next to the proximal part of the rhodopsin staining and that loss of lebercilin led to the disruption of the distal axoneme, the microtubule-based cytoskeleton of the OS. To further investigate the impact of lebercilin loss on OS development, we examined the OS protein rhodopsin over time. We first analyzed rhodopsin and tubulin at low magnification (Supplemental Figure 4). We found a significant decrease in ONL thickness over time, with only a few layers of photoreceptor nuclei left at P28 (Supplemental Figure 4, B and C). Moreover, rhodopsin was mislocalized to the ONL (Supplemental Figure 4, B and D), validating our previous observations (7). To get a better understanding of the rhodopsin mislocalization, we looked at rhodopsin and tubulin at high magnification (Figure 4, A and B). In contrast to CEP290 and RP1, which appeared unchanged at P10, we observed that rhodopsin localization started to be affected at P10, with a clear accumulation of rhodopsin above the basal body (Figure 4B, white arrows), suggesting defective rhodopsin trafficking along the photoreceptor axoneme. At this time point, the rhodopsin staining at the OS level did not seem to be impaired, but this rapidly exacerbated from P14 onward (Figure 4B). Besides the accumulation of rhodopsin above the basal body, we observed rhodopsin along the CC (Figure 4B, white arrowheads) and in membrane vesicle-like structures (Figure 4B, open white arrowheads), further supporting the rhodopsin transport defect hypothesis, which precedes the CC overextension and the distal axoneme disruption.

### Lebercilin regulates IFT-B trafficking at the bulge region.

To investigate the potential trafficking defect, we next investigated the precise localization of the IFT-B protein IFT81 in photoreceptors of HET mice, followed by analyzing the alterations seen in photoreceptors of HOM mice over time. In HET mice, IFT81 accumulated above the basal body and at the bulge region but also to a lesser extent along the CC (Figure 4C). To assess whether IFT81 and lebercilin localized to the same proximal region at the bulge, we measured the distance from the centriolar proximal end to the proximal end of both signals and found that IFT81 and lebercilin localized to the same position in the bulge with the exception of the P18 time point (Figure 4E). Moreover, we found that IFT88, another IFT-B protein, displayed a similar localization at the bulge but also localized at the base of the cilium with a 9-fold symmetry, similarly to what has recently been described in the green algae *Chlamydomonas* (36) (Figure 4H).

Next, we analyzed the effect of lebercilin loss on IFT81 localization. We observed a significant decrease in IFT81 levels above the basal body from P14 onward to P28 (Figure 4, D and F). IFT81 localization was similarly decreased at the bulge at every time point, and we noticed that the signal was dispersed along the distal axoneme before vanishing at P28 (Figure 4, D and G).

Taken together, our results demonstrate that the loss of lebercilin affects the localization of IFT81, which we postulate to result in the mis-trafficking of rhodopsin leading to defective OS membrane disc formation and ultimately photoreceptor cell degeneration.

### Probing LCA5 gene augmentation therapy with U-ExM reveals partial rescue of photoreceptor cells.

Capitalizing on the molecular and structural understanding of the LCA5-associated disease gained using U-ExM, we next decided to probe the AAV-*LCA5* gene augmentation therapy (23, 24) to further improve its efficacy.

Since lebercilin has been found to be expressed 15 days after intra-ocular delivery (23), we focused our analysis on the P18, P22, and P28 time points. Intriguingly, when dissecting the retinas, we noticed that the retina did not fully recover its normal aspect, as seen by the degree of pigmentation, suggesting that the gene augmentation was probably only partial (Supplemental Figure 5A).

**Figure 5:**
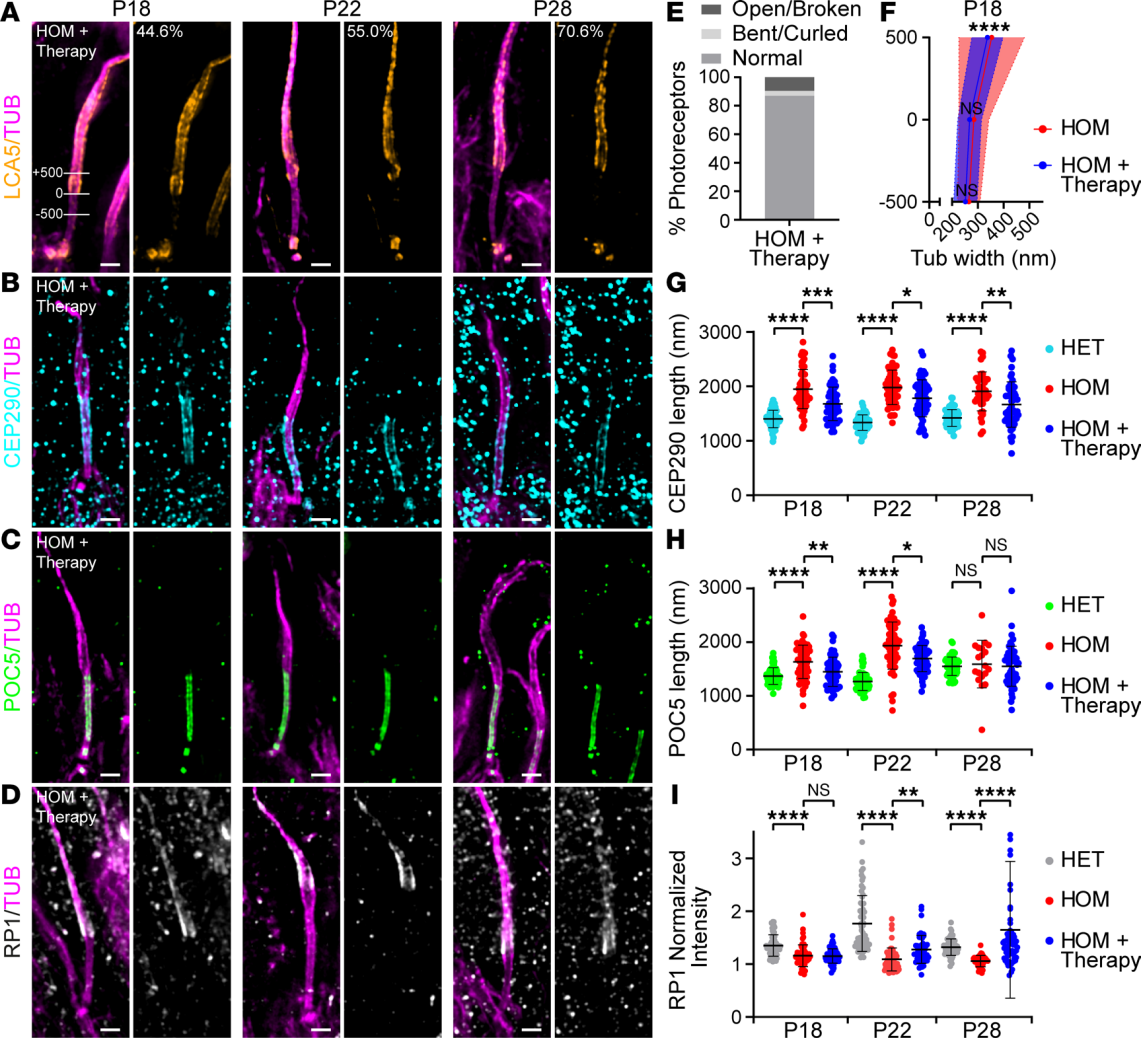
Effect of AAV-*LCA5* gene augmentation therapy on distal axoneme organization and CC length. (**A**–**D**) Widefield (original magnification, 63×) images of expanded photoreceptors stained for tubulin (magenta) and lebercilin (LCA5; orange, **A**), CEP290 (cyan, **B**), POC5 (green, **C**), or RP1 (white/gray, **D**) from P18 to P28 in AAV-*LCA5* gene therapy–treated *Lca5^gt/gt^* mice (HOM + Therapy). Lines in P18 LCA5 image (**A**) illustrate measurements shown in **F** of tubulin width at 3 locations: +500 nm, 0 nm, −500 nm. The proximal end of the LCA5 signal was used to set the 0 location. **A** indicates the percentage of photoreceptors that express LCA5 at each time point (*n* = 109–184). Three animals per time point. Scale bars: 500 nm. (**E**) Distal axoneme (above CC) conformations of HOM + Therapy photoreceptors from P18 to P28 indicated in percentages. Photoreceptor distal axoneme conformations: normal (87%), open/broken (9.6%), and bent/curled (3.4%). *n* = 146. (**F**) Tubulin width measurements of P18 HOM photoreceptors, gene therapy treated versus nontreated, at the 3 locations depicted in **A**. Average tubulin width at each location is indicated by a red or blue dot for HOM and HOM + Therapy, respectively. Only photoreceptors that express LCA5 were used for the measurements. HOM measurements correspond to the data presented in Figure 3C. Three animals per time point. Data presented as mean ± SD; *n* = 27–37. ****P < 0.0001 by *F* test. Significance represents tubulin width dispersion between HOM and HOM + Therapy. (**G**–**I**) Impact of AAV-*LCA5* gene therapy on CEP290 length (**G**), CC inner scaffold length (POC5, **H**), or RP1-normalized intensity at the bulge region (**I**) from P18 to P28. HET and HOM measurements correspond to the data in Figure 3, G–I. Three animals per time point. Data presented as mean ± SD; *n* = 39–65 (**G**), *n* = 19–60 (**H**), *n* = 39–62 (**I**). *P < 0.05, **P < 0.01, ***P < 0.001, ****P < 0.0001 by Kruskal-Wallis test with Dunn’s multiple-comparison test.

Consistently, lebercilin was detected in 44.6%, 55.0%, and 70.6% of P18, P22, and P28 photoreceptors, respectively (Figure 5A). The gradual increase in percentage of lebercilin-positive photoreceptors could possibly be explained by the increasing loss of photoreceptors that were not targeted by AAV-*LCA5* at later time points. At low magnification, ectopic lebercilin expression was observed through all retinal layers (Supplemental Figure 5B). In photoreceptors, lebercilin localization was restored, but not restricted to the bulge region, as it was found along the entire distal axoneme (Figure 5A). Furthermore, lebercilin seemed enriched at the centrioles compared with the endogenous expression of lebercilin (Figure 5A). These results demonstrate that lebercilin ectopic expression, while partially restoring the normal lebercilin localization, also induces unexpected distribution, which might affect the full functional rescue.

Next, we evaluated the effect of *LCA5* augmentation on axonemal photoreceptor axoneme rescue, combining all 3 time points (P18, P22, and P28). Importantly, we found that more than 87% of the lebercilin-positive photoreceptors contained a normal-shaped distal axoneme (Figure 5E). Furthermore, looking at lebercilin-positive photoreceptors, we found that the dispersion in tubulin width above the CC was significantly reduced at P18 in the gene therapy–treated retinas (Figure 5F), indicative of a partial rescue of axonemal defects upon lebercilin expression.

Next, we monitored the effect of the gene augmentation therapy on the distribution of the CC proteins CEP290 and POC5 in the entire photoreceptor population (including the lebercilin-positive and -negative cells). We found that both CEP290 and POC5 signal lengths were significantly reduced in the AAV-*LCA5*–treated retinas (Figure 5, B, C, G and H) but not fully restored to the normal length.

We next tested whether RP1 distribution is restored upon lebercilin expression. We found that RP1 localization was not restored at P18 (Figure 5, D and I). At P22 and P28, however, the RP1 signal at the distal axoneme was significantly rescued compared with the uninjected group (Figure 5, D and I), indicating that exogenous lebercilin expression is sufficient to drive proper RP1 localization.

**Figure 6:**
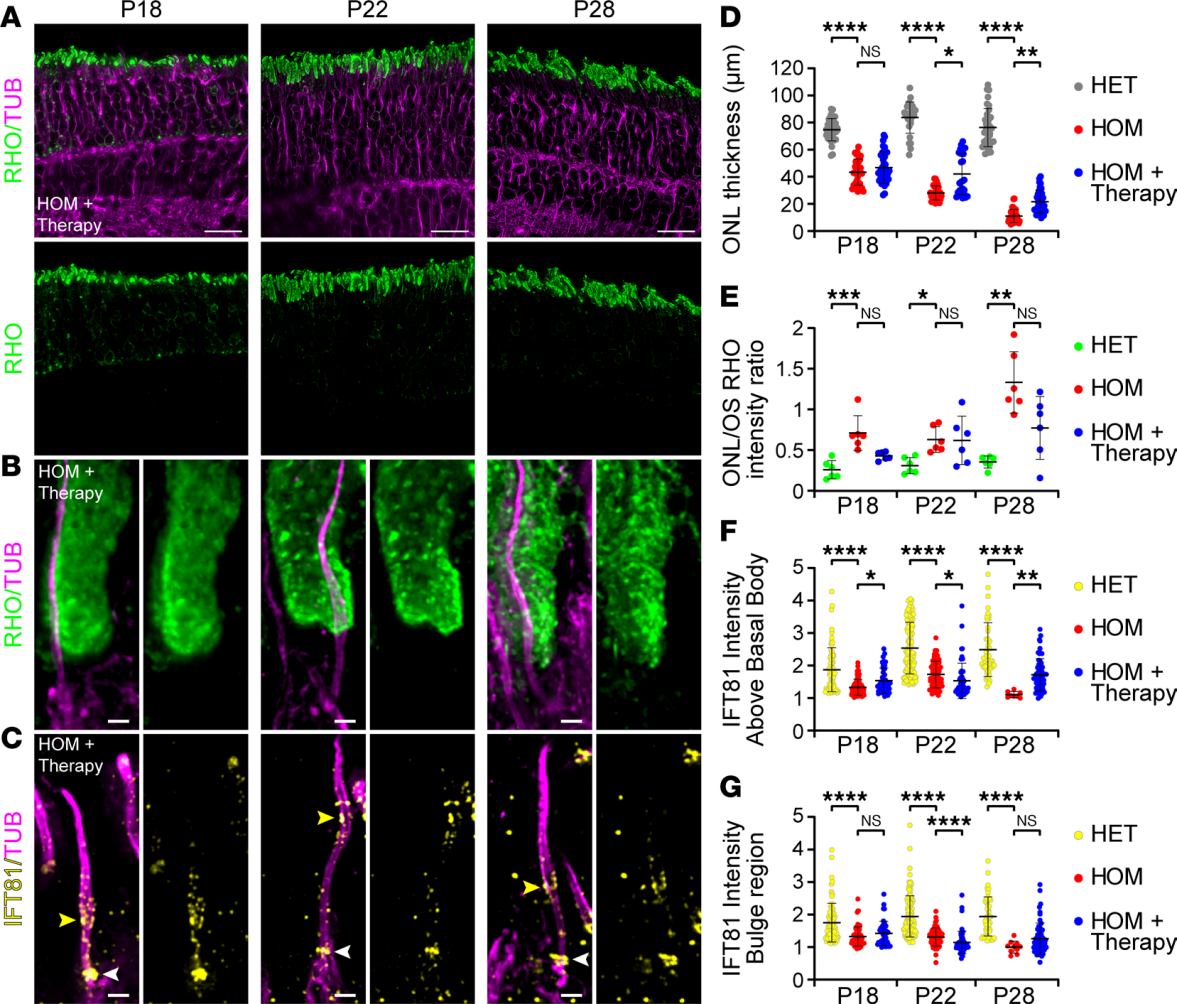
Effect of AAV-*LCA5* gene augmentation therapy on OS formation and intraflagellar transport. (**A**) Low-magnification (original magnification, 20×) widefield images of expanded *Lca5^gt/gt^* (HOM) retinas, treated with AAV-*LCA5* gene therapy (HOM + Therapy), showing rod OS restoration from P18 to P28 by staining with rhodopsin (green) and tubulin (magenta). Scale bars: 20 μm. (**B** and **C**) Widefield (original magnification, 63×) images of expanded photoreceptors stained for tubulin (magenta) and rhodopsin (green, **B**) or IFT81 (yellow, **C**) from P18 to P28 in AAV-*LCA5* gene therapy–treated HOM mice. White arrowheads in **C** indicate IFT81 localization above the basal body. Yellow arrowheads in **C** indicate IFT81 localization at the bulge region. Scale bars: 500 nm. (**D**–**G**) Impact of AAV-*LCA5* gene therapy on outer nuclear layer (ONL) thickness (**D**), ONL/OS rhodopsin intensity ratio (**E**), IFT81-normalized intensity above the basal body (**F**), or IFT81-normalized intensity at the bulge region (**G**) from P18 to P28. Note that HET and HOM measurements in **F** and **G** correspond to data in Figure 4, F and G. Three animals per time point. Data presented as mean ± SD; *n* = 28–36 (**D**), *n* = 6 (**E**), *n* = 8–113 (**F**), *n* = 10–113 (**G**). *P < 0.05, **P < 0.01, ***P < 0.001, ****P < 0.0001 by Kruskal-Wallis test with Dunn’s multiple-comparison test.

Finally, we investigated whether *LCA5* augmentation had an effect on OS restoration and photoreceptor viability. To do so, we first measured the ONL thickness and found a modestly but significantly increased ONL thickness at P22 and P28 (Figure 6, A and D). Moreover, rhodopsin localization seemed partially restored at P18 and P28, with reduced mislocalization at the ONL level, though this was not significant (Figure 6, A and E). Importantly, at high magnification, part of the photoreceptors exhibited total OS restoration, with rhodopsin localizing in a rod-shaped manner and no accumulation above the basal body at all time points (Figure 6B). Despite these promising results, we noticed large heterogeneity in OS restoration between replicates, possibly due to the variability in the injection procedure, the number of targeted photoreceptors, or the lebercilin expression levels (Supplemental Figure 5C).

The above results suggest that rhodopsin trafficking is partially restored by gene therapy. To verify that trafficking is restored, we monitored IFT81 localization at the bulge region and above the basal body. At the bulge region, IFT81 levels were slightly increased after gene therapy at P18 and P28, though this was not significant (Figure 6, C and G). In contrast, IFT81 levels at P22 were reduced by AAV-*LCA5* treatment compared with untreated photoreceptors (Figure 6, F and G), probably due to the heterogeneity of lebercilin expression inside the retina and the ectopic expression along the distal axoneme (Figure 5A and Supplemental Figure 5A). Above the basal body, the levels of IFT81 were significantly increased by the *LCA5* augmentation at P18 and P28 (Figure 6, C and F), suggesting a partial rescue of IFT81, primarily above the basal bodies.

**Figure 7:**
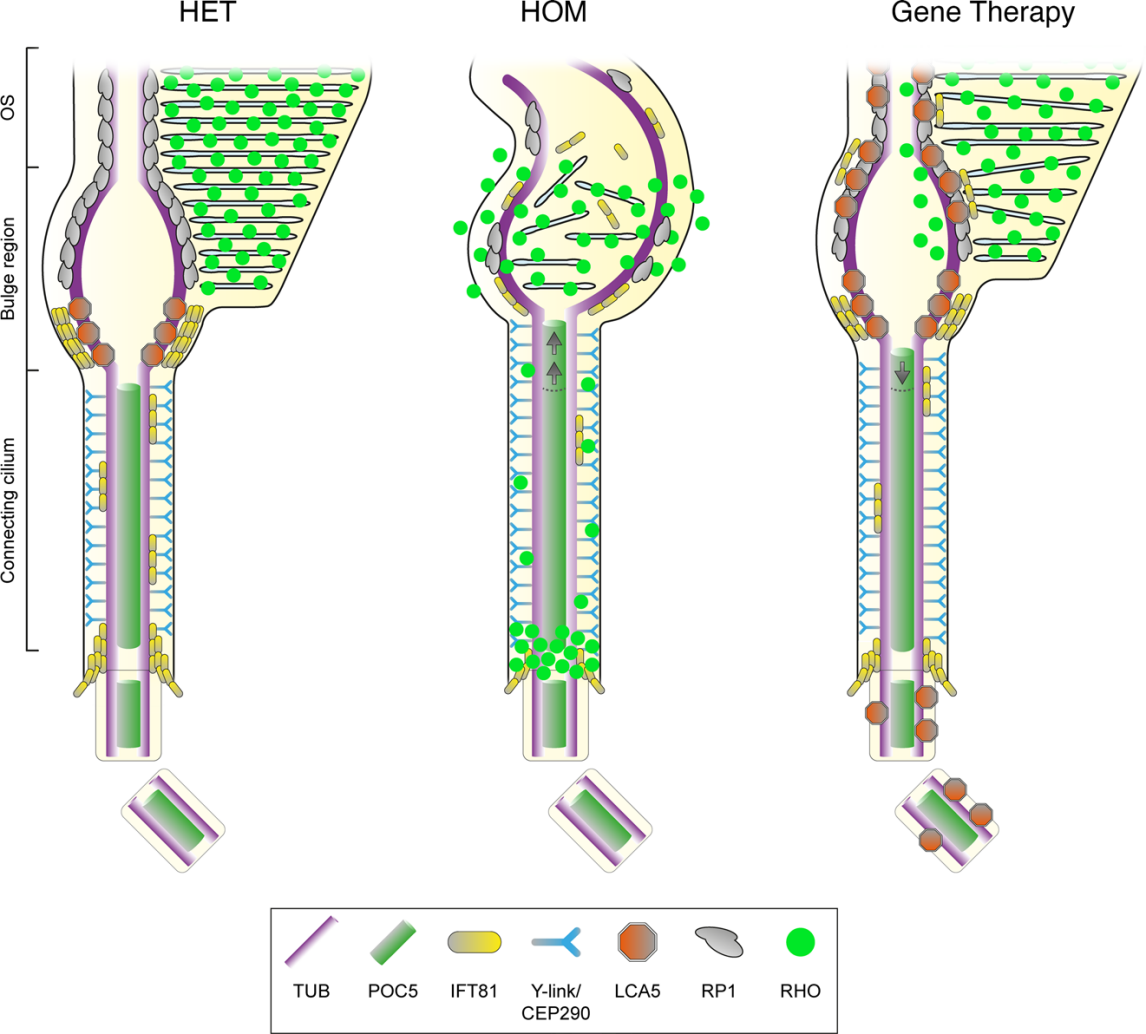
Schematic representation of *Lca5^+/gt^* (HET), *Lca5^gt/gt^* (HOM), and gene therapy–treated photoreceptors. In unaffected HET photoreceptors (left), lebercilin (LCA5; orange) localizes predominantly at the proximal part of the bulge region, between CEP290 (cyan, at the level of the Y-links) and RP1 (gray, distal axonemal protein). IFT81 (yellow) localizes to the same bulge proximal region as LCA5, but also accumulates above the basal body and to a lesser extent along the CC. In HOM photoreceptors (middle), the CC is extended, as illustrated by an elongated CEP290 and POC5 (green) signal. Furthermore, bulge formation and distal axoneme organization is disrupted, leading to rhodopsin (RHO, bright green) mis-trafficking, with accumulation above the basal body, localization along the CC, and inside vesicle-like structures. Moreover, LCA5 loss leads to decreased levels of IFT81 and RP1 at the bulge region and more dispersed localization along the distal axoneme. AAV-*LCA5* gene augmentation therapy (right) partially restores bulge formation, CC and distal axoneme organization, as well as RP1 and rhodopsin localization. IFT81 localization is restored to a lesser extent, possibly explained by the ectopic LCA5 expression along the distal axoneme.

Together, our results illustrate the power of U-ExM to decipher the molecular mechanisms behind lebercilin, as well as to probe the impact at the subcellar level of gene augmentation as a therapeutic strategy (summarized in Figure 7). In particular, our work unveils the function of the bulge region defined by the lebercilin protein, whose depletion led to abnormal axonemal structures, defective IFT and rhodopsin trafficking, and severely affected OS membrane disc formation. We further show that *LCA5* gene augmentation appeared as a promising therapeutical strategy that needs further optimization using, notably, the U-ExM pipeline that we describe in this paper.

## Discussion

The sensory cilium of the photoreceptor cell is one of the longest cilia in the human body, notably due to the stacking of hundreds of opsin-loaded membrane discs. This causes a massive elongation of its tip, the OS, which is required for efficient photoreception and transduction. The importance of this unique sensory organelle is highlighted by the number of retinal diseases associated with mutations in genes coding for OS structure or function (37). The difficulty of characterizing molecular and structural mechanisms involved in photoreceptor degeneration in such diseases comes mostly from the lack of resolution of current imaging tools. To overcome this problem, we recently adapted U-ExM for use in retinal imaging, enabling assessment of the photoreceptor cilium on regular fluorescence microscopes at nanoscale resolution. By this technique, we recently provided a molecular mapping of the OS CC, which helped us understand molecular and structural mechanisms associated with a subtype of retinitis pigmentosa (13).

Here, we continue the molecular characterization of the OS focusing on the bulge region that directly extends from the CC. We first describe that lebercilin decorates MTDs externally on the proximal part of the bulge region. We also show that RP1, a lebercilin interactor revealed by affinity proteomics, localizes immediately distal to lebercilin, extending its presence toward the axoneme distal end. Interestingly, we revealed that the bulge region corresponds to the location where rhodopsin-enriched membrane discs are formed, corroborating recent results suggesting that this region plays a role in the formation of membrane discs via an actin-dependent process involving PCARE and WASF3 proteins (15). Therefore, the bulge region appears as a strategic hub of the OS axoneme, explaining why mutations in genes encoding bulge proteins, such as lebercilin, could have dramatic consequences on photoreceptor development and/or maintenance (1, 7). We previously demonstrated that this region exhibits a typical enlargement of the axonemal MTDs, whose function is yet to be determined. One could imagine that given the intense turnover of membrane discs, the bulge could act as a reservoir to maintain the constant amount of proteins needed to form membrane discs. In line with this, we also showed that the IFT-B machinery accumulates at the level of the bulge region, exactly where lebercilin is located, corroborating the interaction of these 2 modules at the bulge (7, 38, 39). One of the roles of lebercilin at the bulge could be to prevent IFT cargos from reaching the more distal part of the axoneme, thus concentrating OS building blocks brought by IFT at the location where membrane discs form. Finally, given the microtubule-associated feature of lebercilin, we also speculate that it could play a role on the structural maintenance of MTDs at the level of the bulge region.

Consistent with this model, we show that lebercilin loss leads to rapid and drastic disorganization of the OS axoneme above the CC from P14 onward, accompanied by loss of the bulge region. Surprisingly, CC and centriole structure remain unaffected (Figure 3, D and E), but CC length is significantly increased when the bulge is absent (Figure 3, G and H), suggesting that lebercilin could act as a ruler to dictate inner scaffold and CEP290 length. The loss of the bulge structure was accompanied by the loss of bulge-associated proteins, RP1 and IFT81, showing that their localization is dependent on lebercilin. The concomitant loss of IFT81 at the level of the cilium entry could then be the consequence of a lack of protein recycling by IFT when the bulge is lost. In line with this, we show that IFT81 signal at the bulge in HOM mice is progressively spread toward the distal axoneme before being lost, corroborating the role of lebercilin to stop IFT trains at the level of the bulge, allowing them to return to the cell body. The consequent OS collapse could result from the combination of the loss of cohesion between MTDs in the absence of lebercilin, RP1 loss — which has been described to be required for proper OS disc orientation (30, 35) — and IFT defects, possibly preventing the trafficking of OS building blocks.

Our study also explores, with nanoscale resolution, the subcellular outcome of gene therapy using U-ExM. Therefore, our study provides additional critical information in subcellular transgene expression that is lacking in current preclinical gene therapy studies, making our approach a valuable tool that could help further optimize the efficacy of gene augmentation therapy.

In this study, we show that AAV-*LCA5* injection in HOM retina leads to localization of lebercilin inside photoreceptor cells at the expected bulge region, similar to endogenous lebercilin, but also localizes at the entire distal axoneme and the basal body. We argue that this is due to the overexpression of lebercilin, which in AAV-*LCA5* is under control of a strong promoter (CMV) (23). Whether the presence of lebercilin beyond the bulge region and throughout the axoneme could have deleterious effects on OS function remains an open question, but it may not be completely neutral. Importantly, affinity proteomics data from AAV-*LCA5*–injected retina reveal enrichment of several centrosomal proteins, which we suggest to be associated to lebercilin centriolar localization upon ectopic overexpression. Whether these potential interactors could have a role on the *LCA5*-associated phenotype remains to be determined. However, we show that exogenous lebercilin expression in photoreceptors partially rescues axoneme defects, RP1 and IFT protein localization, and CC length and is associated with improved photoreceptor survival. The relatively modest level of rescue could be explained by several arguments. First, the phenotype observed in HOM retina appears very early, between P10 and P14 onward, whereas it has been described that exogenous lebercilin is expressed 15 days after intravitreal delivery (23), performed at P2 in the current study. Second, intravitreal injection of AAV-*LCA5* leads to overexpression of lebercilin in virtually all retina layers, possibly leading to off-target or deleterious effects. Also, the precise expression pattern of lebercilin through the whole area of the retina seems heterogenous, as we noticed different degrees of pigmentation inside the injected retinas. Finally, lebercilin being a microtubule-binding protein, an important local cellular overexpression can impair the microtubule network and lead to cell death. Together, these results show the importance of a precise and controlled spatiotemporal expression of lebercilin to improve gene therapy efficacy for LCA, which already showed interesting functional results, including partial restoration of electroretinograms, amelioration of pupillary light responses, and improved functional vision (23, 24). In the will to improve rescue levels for potential future therapeutic purposes, one possibility could be to perform subretinal injection rather than intravitreal injection to exogenously express lebercilin in the photoreceptors. Indeed, Song and colleagues showed that subretinal injection leads to a better functional outcome compared with intravitreal injection but also to reduced unspecific lebercilin expression in other retina cell layers (23). Combining this injection method with a photoreceptor-specific promoter could then optimize the expression levels of lebercilin in a spatially controlled manner and thus increase the level of rescue. Furthermore, subretinal injection leads to rapid expression of the transgene in a few days (40), which would represent a crucial advantage for early-onset diseases such as LCA. Finally, a detailed analysis of the subcellular pattern of lebercilin expression using U-ExM could help corroborate functional rescue experiments, crucial for future translational studies. With U-ExM, we now have the spatial resolution to accurately monitor rescue or induction of subcellular phenotypes, such as axoneme reformation or recruitment of interactors. U-ExM represents a powerful tool to assess and improve gene therapy efficacy in many cellular or tissue contexts, which are crucial steps to design preclinical studies before a clinical trial can be initiated.

## Methods

### Mouse model

The *Lca5^gt/gt^* (HOM) and *Lca5^+/gt^* (HET) mice (C57BL/6J [B6] background; P. Nishina, University of Tübingen, Tübingen, Germany) (7) were maintained at room temperature (RT) with a 12-hour light/12-hour dark cycle with light on at 7 am and were fed ad libitum. For affinity proteomics, mice were sacrificed at about P28 and retinas were harvested. For U-ExM, mice were sacrificed at P10, P14, P18, P22, and P28.

### Intravitreal injections

Intravitreal (IVT) injections were performed similarly as described before (41). Briefly, P2 HOM and HET mouse pups of either sex were anesthetized by hypothermia. Eyelids were opened using a 30-gauge needle. An incision was made into the sclera at the nasal part of the retina. For affinity proteomics, 0.6 μL of AAV7m8.CbA.hopt-*LCA5* (9.87 × 10^9^ viral genomes (vg)/μL; J. Bennett, University of Pennsylvania, Philadelphia, Pennsylvania, USA) (23), containing a C-terminal 3xFLAG-tag, was injected in one eye using a Hamilton syringe, while 0.6 μL of AAV7m8.CbA.*eGFP* (control vector; 9.98 × 10^9^ vg/μL; J. Bennett, University of Pennsylvania, Philadelphia, Pennsylvania, USA) (23) was injected in the other eye as an internal control. For U-ExM, 0.6 μL of AAV7m8.CbA.hopt-*LCA5* (9.87 × 10^9^ vg/μL) (23), containing a C-terminal 3xFLAG-tag, was injected in one eye using a Hamilton syringe, while the other eye was left untreated to not interfere with the morphological development.

For affinity proteomics, a total of 15 mice for each genotype (HOM and HET) were injected per biological replicate (*n* = 5). For U-ExM, a total of 15 mice for each genotype (HOM and HET) were injected, using 3 mice of each genotype per time point (P10, P14, P18, P22, and P28).

### Affinity proteomics on mouse retina

Four weeks after IVT injections, retinas were harvested by making a cut in the eye and removing the lens, followed by carefully squeezing out the retina with a forceps. Harvested retinas were pooled per group (HOM + AAV-*LCA5*; HOM + AAV-*eGFP*; HET + AAV-*LCA5*; HET + AAV-*eGFP*). Retinas were lysed in lysis buffer containing 0.5% Nonidet-P40 [NP-40], protease inhibitor cocktail (Roche), and phosphatase inhibitor cocktails II and III (MilliporeSigma) in TBS (30 mM Tris-HCl, pH 7.4, and 150 mM NaCl) for 30 minutes at 4°C, followed by high-speed shaking (30 Hz) using Tissuelyser II (QIAGEN) and sonication (>20 kHz; 10 cycles: 30 seconds on/30 seconds off) at 4°C. Lysates were cleared by centrifugation at 10,000*g* for 15 minutes at 4°C. Cleared lysates with equal amounts of proteins were transferred to anti-FlagM2 affinity gel (MilliporeSigma) and incubated for 1.5 hours at 4°C. Subsequently, the affinity gel with bound protein complexes were washed 3 times with wash buffer (TBS containing 0.1% NP-40 and phosphatase inhibitor cocktails II and III), followed by 2 times with 1× TBS. On-bead digestion was performed for 30 minutes at 27°C and 800 rpm, in trypsin digestion buffer (2 mM urea, 50 mM Tris-HCl at pH 7.5, 5 μg/sample trypsin from Serva, 37283). The supernatant was collected and the beads were washed once with urea/DTT buffer (2 mM urea, 50 mM Tris-HCl at pH 7.5, 1 mM DTT). The on-bead digestion and wash were pooled per sample and incubated overnight (ON) at RT. The next day, samples were snap-frozen and stored at –80°C until mass spectrometry analysis.

### Mass spectrometry analysis, protein quantification, and statistics

The trypsinized protein samples were subjected to iodacetamide (5 mg/mL, Merck, 8.04744.0025) and StageTip purified (Thermo Fisher Scientific), followed by mass spectrometry analysis on an Orbitrap Fusion Tribrid mass spectrometer (Thermo Fisher Scientific) as described earlier (39). Label-free quantification was performed, using Maxquant (v.1.6.1.0) (42, 43). For peptide and protein identification, the following subset of the SwissProt database was used: mouse release 2019_09, 17,021 entries. This procedure was performed twice per sample and combined in one data set using the mean values. Identified proteins were analyzed using Perseus (v1.6.2.3). All data were filtered for potential contaminants, peptides only identified by site, and reverse database identifications. The proteins were filtered to be present in at least 3 of the 5 replicates. For AAV-*LCA5*– versus AAV-*eGFP*–injected (control) retinas, a 1-sided 2-sample test was performed (*P* < 0.05). Furthermore, a significance A outlier test with permutation-based FDR correction was performed (FDR < 0.05). Proteins were considered significantly enriched when passing both the 2-sample test (*P* < 0.05) and the significance A outlier test (FDR < 0.05). Data sets from HOM and HET mice were analyzed separately.

### U-ExM on mouse retinas

Retinas were prepared for U-ExM, as described earlier (13). In short, eyes of P10, P14, P18, P22, and P28 mice were enucleated and fixed for 15 minutes at RT in 4% paraformaldehyde/PBS. Subsequently, the cornea and lens were removed with micro-scissors followed by carefully removing the retina. The retinas were incised to flatten and placed inside a 10 mm microwell of a 35 mm Petri dish (P35G-1.5-10-C, MatTek) for U-ExM processing.

The expansion procedure is an adaptation of the original U-ExM protocol that we have optimized for eye tissue samples as previously described (13, 44). Briefly, retinas were incubated ON in 100 μL of 2% acrylamide (AA; A4058, MilliporeSigma) + 1.4% formaldehyde (FA; F8775, MilliporeSigma) at 37°C. The next day, retinas were incubated in 35 μL monomer solution composed of 25 μL of sodium acrylate (stock solution at 38% [w/w] diluted with nuclease-free water, 408220, MilliporeSigma), 12.5 μL of AA, 2.5 μL of N,N′-methylenebisacrylamide (BIS, 2%, M1533, MilliporeSigma), and 5 μL of 10× PBS for 90 minutes at RT. Subsequently, the monomer solution was removed, and retinas were incubated in 90 μL of monomer solution with the addition of ammonium persulfate (APS, 17874, Thermo Fisher Scientific) and tetramethylethylenediamine (TEMED, 17919, Thermo Fisher Scientific) as a final concentration of 0.5% for 45 minutes at 4°C followed by 3 hours’ incubation at 37°C. A 24 mm coverslip was added on top to close the chamber. Next, the coverslip was removed, and 1 mL of denaturation buffer (200 mM SDS, 200 mM NaCl, 50 mM Tris base in water at pH 9) was added into the MatTek dish for 15 minutes at RT with shaking to detach the gel from the dish. Afterward, the gel was incubated in denaturation buffer for 1 hour at 95°C followed by ON at RT. The next day, the gel was sliced around the retina that was still visible at this step, then expanded in 3 consecutive double-distilled (dd) H_2_O baths. Next, the gel was manually sliced with a razor blade to obtain approximately 0.5 mm thick transversal sections of the retina to enable processing for immunostaining.

For immunostaining, commercial or custom (7, 31, 45) primary antibodies (Supplemental Table 2) were incubated ON at 4°C or for 3 hours at 37°C for anti-RP1 (Q. Liu and R. Butcher, Harvard Medical School, Boston, Massachusetts, USA). Image acquisition was performed on an inverted Leica Thunder small volume computational clearing mode at max resolution, adaptive as “strategy” and water as “mounting medium” to generate denoised images. *Z*-stacks were acquired with 0.21 μm *Z* intervals and an *X*,*Y* pixel size of 100 nm.

### Human cell culture and expansion

U2OS cells (ATCC) were grown at 37°C with 5% CO_2_ in DMEM supplemented with GlutaMAX (Life Technologies), 10% fetal calf serum (Brunschwig), and penicillin and streptomycin (100 μg/mL). For U-ExM, U2OS cells were plated onto coverslips in a 6-well plate at 300,000 cells/well and processed 24 hours later. Briefly, coverslips were incubated for 3 hours in 100 μL of 2% AA (A4058, MilliporeSigma) + 1.4% FA (F8775, MilliporeSigma) at 37°C. Then, coverslips were incubated in monomer solution (10% AA, 19% sodium acrylate, 0.1% BIS in 1× PBS) containing 0.5% TEMED and APS for 5 minutes on ice followed by 1 hour at 37°C. Next, the gel was detached from the coverslip by adding 1 mL of denaturation buffer (200 mM SDS, 200 mM NaCl, 50 mM Tris base in water at pH 9) for 15 minutes under shaking into a well of a 6-well plate. The gel was then transferred to a 1.5 mL tube and incubated in denaturation buffer for 1.5 hours at 95°C. Gels were washed from the denaturation buffer twice in ddH_2_O. Then, gels were shrunk in 1× PBS and stained for 3 hours at 37°C in 1× PBS-BSA 2% for both primary (Supplemental Table 2) and secondary antibodies (anti-mouse 488: A11029; anti-mouse 568: A11004; anti-rabbit 488: A11008; anti-rabbit 568: A11036; anti-chicken 568: A11041; Thermo Fisher Scientific; 1:400), followed each by 3 washes for 5 minutes with 1× PBS–Tween 0.1%. Finally, gels were next re-expanded in ddH_2_O and imaged.

### Quantifications

#### Expansion factor.

The expansion factor was calculated in a semiautomated way by measuring the full width at half maximum (FWHM) of photoreceptor mother centriole proximal tubulin signal using PickCentrioleDim plugin, as described before (13). A total of 100 photoreceptor mother centrioles, divided over both genotypes, all time points, and all 3 replicates, were subjected to FWHM measurements and compared with a preassessed value of U2OS centriole width (mean = 231.3 nm ± 15.6 nm) (13). Calculating the ratio between measured FWHM and known centriole width resulted in an expansion factor of 4.39 (Supplemental Table 3). This expansion factor was then used for every quantification. Scale bars shown in all figures are corrected for the expansion factor.

#### Protein signal length and position.

Protein signal lengths or position compared with basal body proximal end (depicted with tubulin) were measured using a segmented line drawn by hand (FIJI) to fit with photoreceptor curvature and corrected with the expansion factor. Only photoreceptors where both protein signal (POC5, CEP290, lebercilin, RP1, or IFT81) and basal body proximal end (tubulin) were clearly visible were selected for measurement.

#### Tubulin spread.

Tubulin spread was assessed at P10, P14, P18, and P22 in noninjected retinas and at P18 in injected retinas in a semiautomated way by measuring FWHM of tubulin signal with PickCentrioleDim plugin (13) on 3 locations of the photoreceptor corresponding to 500 nm proximally to the end of the CC POC5 staining (−500), at the level of the end of the CC POC5 staining (0 nm), or 500 nm distally to the end of the CC POC5 staining (+500). For injected photoreceptors, the proximal extremity of the expressed lebercilin signal was used to set the 0 location. Each measurement was subsequently corrected for the expansion factor.

#### Fluorescence intensity.

Fluorescence intensity measurements of RP1, rhodopsin, and IFT81 were performed on maximal projections using FIJI (46) on denoised images. The same rectangular region of interest (ROI) drawn by hand was used to measure the mean gray value of the protein signal and the corresponding background. Fluorescence intensity was finally calculated by dividing the mean gray value of the fluorescence signal by the mean gray value of the background (normalized mean gray value). For RP1, measurements were performed on the bulge region, defined by tubulin. For rhodopsin, measurements were performed on the ONL and on the OS layer on 20× original magnification images. For each image, 3 ROIs were measured for ONL and OS layers, and fluorescence intensity was calculated by dividing average ONL mean gray value by OS average mean gray value. For IFT81, measurements were performed on the region just above the basal body and on the bulge region, defined by tubulin.

#### Axonemal defects.

Axonemal defects were categorized as follows. Photoreceptors showing axoneme bending over 180° were classified as “bent/curled” whereas photoreceptors exhibiting microtubule spreading or even loss of distal axoneme were classified as “open/broken.”

#### ONL thickness.

ONL thickness was measured manually using tubulin and/or DAPI staining on 2 different 20× original magnification images per replicate. Three measurements were performed per image to avoid bias due to retina dissection. Each measurement was subsequently corrected for the expansion factor.

#### Exogenous lebercilin expression in injected retinas.

The percentage of photoreceptors expressing lebercilin was quantified manually from 63× original magnification images, independent of the level of expression.

### Statistics

The comparison of 2 groups was performed using the nonparametric Mann-Whitney *U* test, if normality was not granted because it was rejected by Pearson’s test. The comparison of 3 groups was performed using the nonparametric Kruskal-Wallis test with Dunn’s multiple-comparison test, if normality was not granted because it was rejected by Pearson’s test. Every measurement was performed on 3 animals. Data are all represented as a scatter dot plot with center line as mean, except for percentage quantifications, which are represented as histogram bars. For tubulin width measurements, the variances of each condition (HET, HOM, HOM + Therapy) and at every location (–500, 0, 500) were compared using an *F* test. The graphs with error bars indicate ±SD, and the significance level is denoted as usual (nonsignificant *P* > 0.05, *P < 0.05, **P < 0.01, ***P < 0.001, ****P < 0.0001). All the statistical analyses were performed using GraphPad Prism 9. Every mean, SD, test, and number of animals used for comparison are referenced in Supplemental Table 3. When possible, a minimum of 10 measurements has been performed per animal. The data underlying the graphs shown in all the figures are included in Supplemental Table 3.

### Study approval

*Lca5^gt/gt^* (HOM) and *Lca5^+/gt^* (HET) mice (7) were handled in accordance with the statement of the Animals in Research Committee of the Association for Research in Vision and Ophthalmology, and experiments were approved by the Animal Ethics Committee of the Radboud University Medical Center (AVD10300 2016 758; RU-DEC-2016-0050).

## Author contributions

SF and OM participated in experimental design; performed research; collected, analyzed, and interpreted data; performed statistical analysis; and drafted and revised the manuscript. SF and OM share the first author position, with SF listed first since SF and RR initiated the project. KJ processed samples after affinity purification to perform mass spectrometry analysis. AG assisted in the intravitreal injections, co-supervised the project, and revised the manuscript. HMS assisted in intravitreal injections. MU was responsible for the funding acquisition and supervision of the mass spectrometry analysis. RWJC supervised the project, interpreted data, and revised the manuscript. KB supervised the mass spectrometry analysis, analyzed and interpreted data, and revised the manuscript. PG, VH, and RR were responsible for funding acquisition and supervision of the project, interpreted data, and revised the manuscript. PG, VH, and RR share the last author position, with RR as last author since RR initiated the project. The position of PG and VH was determined alphabetically.

## Supplementary Material

Supplemental data

Supplemental table 1

Supplemental table 3

## Version 1 04/18/2023

In-Press Preview

## Version 2 05/22/2023

Electronic publication
