# Supplementary material for: Gene augmentation of LCA5-associated Leber congenital amaurosis ameliorates bulge region defects of the photoreceptor ciliary axoneme
Source: JCI Insight. 2023 May 22;8(10):e169162. doi: 10.1172/jci.insight.169162 (PMC10322687; doi:10.1172/jci.insight.169162)
Supplement: Supplemental data [file jciinsight-8-169162-s008.pdf]

A

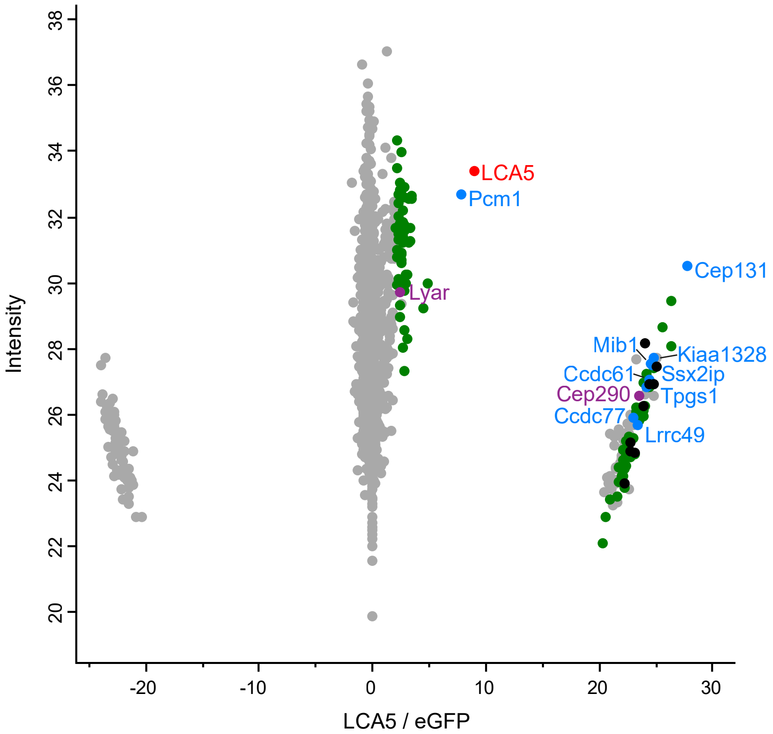

B

Category	Protein names
Photoreceptor proteins	Cep290 Lyar
Centriolar satellite/ Centrosomal proteins	Ccdc61 Ccdc77 Cep131 Kiaa1328 Lrrc49 Mib1 Pcm1 Ssx2ip Tpgs1
Ribonucleo-proteins	[Supplemental Table 1]
Miscellaneous	[Supplemental Table 1]

Supplemental Figure 1. Identification and clustering of potential lebercilin interactors. (**A**) Scatterplot showing enriched proteins comparing AAV-*LCA5* injected retinas to AAV-*eGFP* (control) injected retinas in unaffected *Lca5*$^{+/gt}$ (HET) mice. The bait protein lebercilin (LCA5) is shown in red. Significantly enriched proteins (P<0.05 by Student's t-test and *FDR*<0.05 by significance A test) are categorized into different groups based on their function, including photoreceptor-associated proteins (purple), centriolar satellite/ centrosomal proteins (blue), ribonucleoproteins (green), and miscellaneous (black). X-axis represents log2 ratio between AAV-*LCA5* and AAV-*eGFP* (control) injected retinas. Y-axis represents the intensity score, indicating the relative amount of proteins in the dataset. 15 mice per biological replicate (n=5). (**B**) Table showing the significant proteins categorized in different groups, based on their function. Original data is listed in Supplemental Table 1.

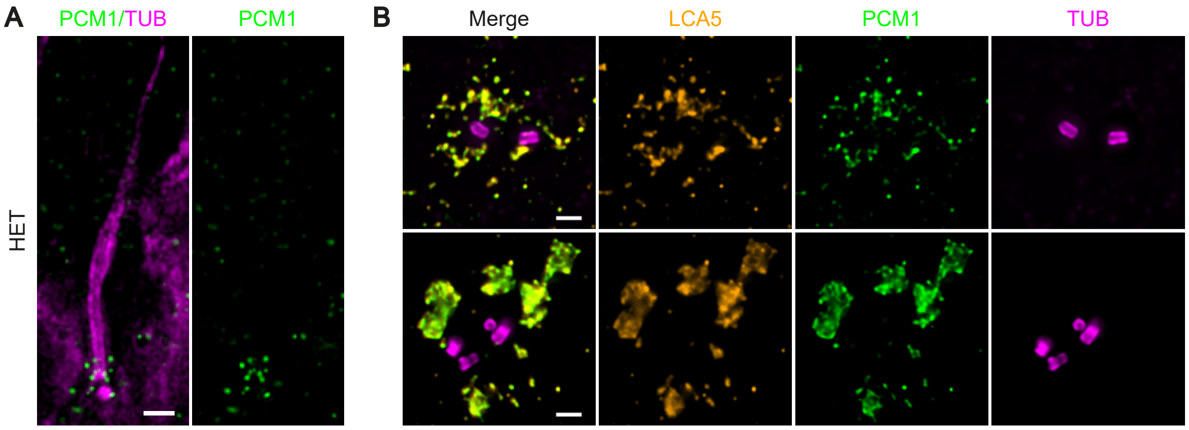

Supplemental Figure 2. Localization of PCM1 in photoreceptor cells and human U2OS cultured cells. (**A**) Widefield (63x) images of expanded adult photoreceptor stained for tubulin (magenta) and PCM1 (green). Scale bar: 500 nm. (**B**) Representative widefield (63x) images of expanded U2OS centrioles stained with tubulin (magenta), lebercilin (LCA5; orange), and PCM1 (green). Scale bars: 500 nm.

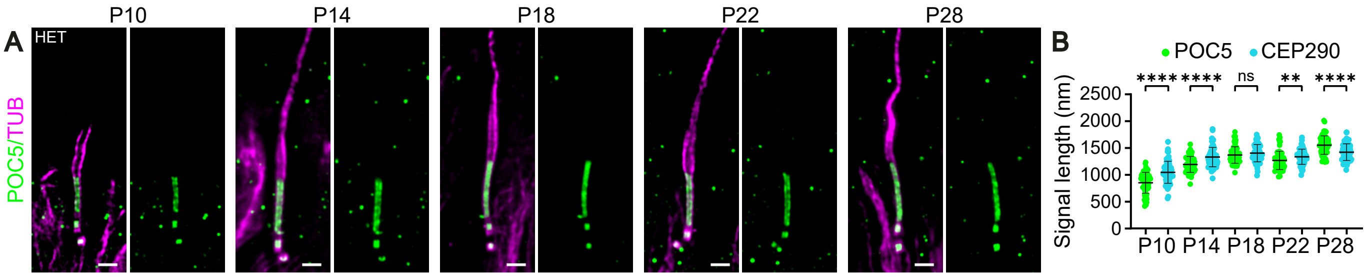

Supplemental Figure 3. Localization of POC5 in developing photoreceptors. (**A**) Widefield (63x) images of expanded photoreceptors stained for tubulin (magenta) and POC5 (green) from P10 to P28 in *Lca5*$^{+/gt}$ (HET) mice. Scale bars: 500 nm. (**B**) Comparison of POC5 (green) and CEP290 (cyan) signal length from P10 to P28 in HET mice. Note that these measurements correspond to the data in Figure 3, G and H. Three animals per timepoint. Data presented as mean ± SD; *n*=60. ***P*<0.01, *****P*<0.0001 by Mann-Whitney test.

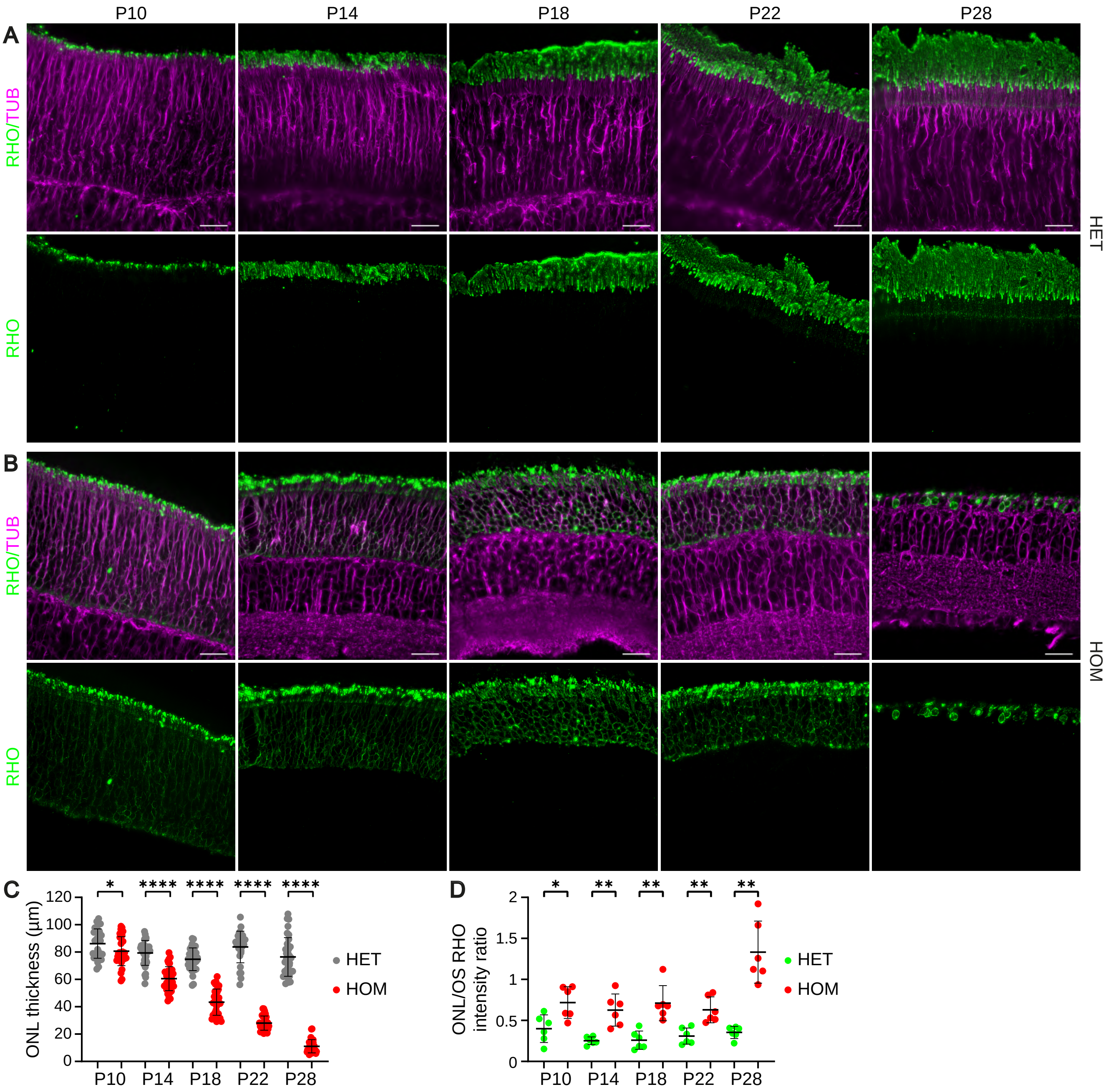

Supplemental Figure 4. Development of retinal layers in *Lca5*$^{+/gt}$ (HET) and *Lca5*$^{gt/gt}$ (HOM) mice. (**A-B**) Low magnification (20x) widefield images of expanded retinas in HET mice (A) and HOM mice (B) showing rod OS development from P10 to P28 by staining with Rhodopsin (green) and tubulin (magenta). Scale bars: 20 µm. (**C-D**) Impact of lebercilin loss on outer nuclear layer (ONL) thickness (C) or ONL/OS Rhodopsin intensity ratio (D) from P10 to P28. Note that the HET and HOM measurements of P18, P22, and P28 correspond to the data in Figure 6, D and E. Three animals per timepoint. Data presented as mean ± SD; *n*=28-36 (C), *n*=6 (D). **P*<0.05, ***P*<0.01, *****P*<0.0001 by Mann-Whitney test.

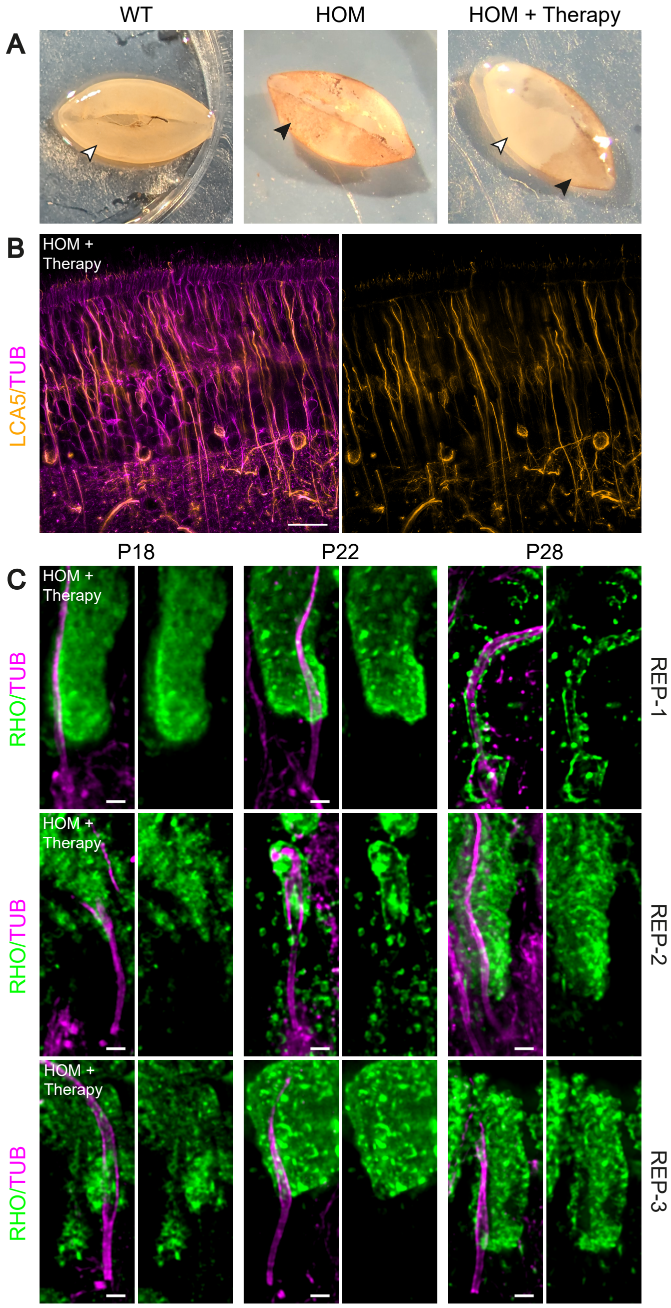

Supplemental Figure 5. Global effect of AAV-*LCA5* gene augmentation therapy. (**A**) Representative pictures of dissected retinas from wild-type (WT), *Lca5*$^{gt/gt}$ (HOM), and AAV-*LCA5* gene therapy treated HOM mice (HOM + Therapy). Lack of pigmentation in WT and HOM + therapy retinas is indicated by open arrowheads and pigmentation in HOM and HOM + therapy retinas is indicated by closed arrowheads. (**B**) Low magnification (20x) widefield images of expanded P28 retina, treated with AAV-*LCA5* gene therapy and stained for lebercilin (LCA5; orange) and tubulin (magenta). Scale bar: 20 µm. (**C**) Representative widefield (63x) images of expanded photoreceptors of each replicate (REP1-3) stained for tubulin (magenta) and Rhodopsin (green) from P18 to P28 in AAV-*LCA5* gene therapy treated HOM mice. Of note: Each replicate represents a different mouse. Scale bars: 500 nm.

Supplemental Table 1. Protein list of potential lebercilin interactors. (**A**) Table showing significantly enriched proteins in AAV-*LCA5* injected retinas compared to AAV-*eGFP* (control) injected retinas in *Lca5*$^{gt/gt}$ (HOM) mice (**Tab 1**) and unaffected *Lca5*$^{+/gt}$ (HET) mice (**Tab 2**). The bait protein lebercilin (LCA5) is shown in red. Significantly enriched proteins are categorized into different groups based on their function, including photoreceptor-associated proteins (purple), centriolar satellite/centrosomal proteins (blue), ribonucleoproteins (green), and miscellaneous (black).

Supplemental Table 2. Antibody list.

Name	Provider	Reference	Dilution
α-Tubulin	Abcam	Ab18251	1:500
α-Tubulin	ABCD antibodies	AA345 - scFv-F2C; Ref 44	1:250
β-Tubulin	ABCD antibodies	AA344 - scFv-S11B; Ref 44	1:250
CEP290	Proteintech	22490-1-AP	1:250
IFT81	Proteintech	11744-1-AP	1:250
IFT88	Proteintech	13967-1-AP	1:250
LCA5	Custom	SN2135; Ref 7	1:250
PCM1	Santa Cruz	Sc-67204	1:250
POC5	Bethyl	A303-341A	1:250
Rhodopsin	ThermoFisher	MA5-11741	1:1000
RP1	Custom	Ref 31	1:250

Supplemental Table 3. Descriptive statistics for all measurements.
